# Offline effects of transcranial direct current stimulation on reaction times of lower extremity movements in people after stroke: a pilot cross-over study

**DOI:** 10.1186/s12984-019-0604-y

**Published:** 2019-11-07

**Authors:** Milou J. M. Coppens, Wouter H. A. Staring, Jorik Nonnekes, Alexander C. H. Geurts, Vivian Weerdesteyn

**Affiliations:** Department of Rehabilitation, Donders Institute for Brain, Cognition and Behaviour, Radboud University Medical Center, PO Box 9101, 6500 HB Nijmegen, The Netherlands

**Keywords:** Transcranial direct current stimulation, tDCS, Stroke, Balance, Posture, Gait, Reaction times

## Abstract

**Background:**

Transcranial direct current stimulation (tDCS) is a non-invasive brain stimulation technique that has shown promise for rehabilitation after stroke. Ipsilesional anodal tDCS (a-tDCS) over the motor cortex increases corticospinal excitability, while contralesional cathodal tDCS (c-tDCS) restores interhemispheric balance, both resulting in offline improved reaction times of delayed voluntary upper-extremity movements. We aimed to investigate whether tDCS would also have a beneficial effect on delayed leg motor responses after stroke. In addition, we identified whether variability in tDCS effects was associated with the level of leg motor function.

**Methods:**

In a cross-over design, 13 people with chronic stroke completed three 15-min sessions of anodal, cathodal and sham stimulation over the primary motor cortex on separate days in an order balanced across participants. Directly after stimulation, participants performed a comprehensive set of lower-extremity tasks involving the paretic tibialis anterior (TA): voluntary ankle-dorsiflexion, gait initiation, and backward balance perturbation. For all tasks, TA onset latencies were determined. In addition, leg motor function was determined by the Fugl-Meyer Assessment – leg score (FMA-L). Repeated measures ANOVA was used to reveal tDCS effects on reaction times. Pearson correlation coefficients were used to establish the relation between tDCS effects and leg motor function.

**Results:**

For all tasks, TA reaction times did not differ across tDCS sessions. For gait initiation and backward balance perturbation, differences between sham and active stimulation (a-tDCS or c-tDCS) did not correlate with leg motor function. Yet, for ankle dorsiflexion, individual reaction time differences between c-tDCS and sham were strongly associated with FMA-L, with more severely impaired patients exhibiting slower paretic reaction times following c-tDCS.

**Conclusion:**

We found no evidence for offline tDCS-induced benefits. Interestingly, we found that c-tDCS may have unfavorable effects on voluntary control of the paretic leg in severely impaired patients with chronic stroke. This finding points at potential vicarious control from the unaffected hemisphere to the paretic leg. The absence of tDCS-induced effects on gait and balance, two functionally relevant tasks, shows that such motor behavior is inadequately stimulated by currently used tDCS applications.

**Trial registration:**

The study is registered in the Netherlands Trial Register (NL5684; April 13th, 2016).

## Introduction

Transcranial direct current stimulation (tDCS) is a non-invasive brain stimulation technique that has shown promise for improving motor control of the paretic limb in people with stroke [1–3]. Anodal tDCS (a-tDCS) over the primary motor cortex (M1) increases corticospinal excitability, while cathodal tDCS (c-tDCS) reduces corticospinal excitability [4]. In people with stroke, corticospinal excitability in the lesioned hemisphere is often reduced, and application of a-tDCS over the affected M1 may thus improve its motor output. Indeed, several studies have shown that offline a-tDCS over the lesioned hemisphere improves motor output to the upper extremity in patients with stroke [5].

The reduced excitability of the lesioned hemisphere may be explained by an imbalance in interhemispheric control [6, 7], with excessive inhibition from the contralesional hemisphere limiting motor output of the lesioned hemisphere. Downregulation of the contralesional hemisphere by c-tDCS has been suggested to restore interhemispheric balance, resulting in improved reaction times of delayed voluntary upper-extremity movements in patients with stroke [8]. Beneficial effects of ipsilesional a-tDCS and contralesional c-tDCS have also been demonstrated during functional task performance, as shown by improvements in the Action Research Arm Test (ARAT) [1] and Jebsen–Taylor Hand Function Test [9, 10]. In addition, recent literature has suggested that c-tDCS would be particularly beneficial to accelerate reaction times during wrist flexion of patients with good motor function (i.e., a relative high score on the Fugl-Meyer Assessment – arm score) [11].

Until now, a limited number of studies have investigated whether these promising results of tDCS also pertain to the lower extremity in patients with stroke. Some studies have shown that a-tDCS over the primary motor cortex can reduce motor evoked potential (MEP) latencies and increase MEP amplitude in the tibialis anterior (TA) muscle of healthy participants [12] and people after stroke [13], whereas c-tDCS over the contralateral M1 had no effects on these MEP parameters [12]. In line with the reported changes in MEP latencies and amplitudes, knee extensor force improved after a-tDCS in both healthy participants and people after stroke [14, 15]. Although gains in force production and MEP have been described, gains in reaction time during voluntary movement, as observed for the upper extremity, have not been found [16, 17]. The mixed effects of tDCS on measures of corticospinal excitability [18, 19] also pertain to clinical outcomes measures [3, 20]. These disparate results call for further research, including concurrent assessments of measures of corticospinal excitability and measures of functional task performance involving the lower extremity. In addition, there is often substantial inter-individual variability in tDCS-induced changes in people with stroke, but it remains to be identified whether a good response to tDCS in lower-extremity tasks is related to clinical characteristics, such as the level of leg motor function.

In the present study, we aimed to determine the offline effects of a-tDCS over M1 of the lesioned hemisphere, and cathodal tDCS over contralesional M1 in people in the chronic phase after stroke, using a comprehensive set of lower-extremity tasks. Offline tDCS effects were assessed as it has the potential to be applied as an adjunct to physical therapy and is commonly studied in people after stroke [3, 20]. We assessed reaction times of TA during voluntary ankle dorsiflexion, gait initiation, and following backward balance perturbation. In healthy adults, a previous study from our group showed that a-tDCS over M1 resulted in accelerated TA reaction times during ankle dorsiflexion and balance perturbations (within 30 min post stimulation) [21]. Here, we expected to find similar speeding up of reaction times in people with stroke, particularly because the previously reported delay in paretic TA reaction times in the selected tasks leaves sufficient room for improvement [22, 23]. We also aimed to investigate whether the potential beneficial offline effects of tDCS would translate into better task performance. Furthermore, we explored whether individual differences in tDCS effects on TA reaction times and task performance would be associated with the level of leg motor function.

## Methods

### Participants

In this study, thirteen people (62 ± 11.6 years; one female) participated who were in the chronic phase (> 6 months) after a unilateral supratentorial stroke. Participants were recruited from local practitioners and patient associations. Participants had to be able to stand independently on bare feet for at least 15 min and take a few steps without a walking aid, and needed to have (corrected to) normal vision and hearing. We excluded participants if they had any other neurological or motor disorder, had evident cognitive impairment (Mini-Mental State Examination score < 24), or used medication that could influence balance control or cortical excitability (e.g. neuroleptics, anti-epileptics and benzodiazepines). For safety reasons regarding tDCS, participants were also excluded if they had large ferromagnetic metal parts or active implants in their upper body, have had brain surgery in the past, had tinnitus, or were pregnant. In addition, participants were asked to consume their regular amount of caffeine, not to smoke more than five cigarettes on the day of the experiment (if any), and not to take any recreational drugs or alcohol 24 h prior to the experiment. Approval for the study was obtained by the medical ethical committee (CMO) region Arnhem-Nijmegen and the study was conducted in accordance with the Declaration of Helsinki. All subjects gave their written informed consent prior to the experiment.

### Study design

Participants visited the lab for one intake session and three tDCS sessions in a period of 5 weeks. During the intake session, we conducted a set of clinical assessments to characterize our study population. The Fugl-Meyer Assessment – leg score (FMA-L) was used to determine motor function (i.e., selective motor control) of the paretic leg [24]. The Motricity Index was used to determine muscle strength of the paretic leg [25]. Balance capacity was assessed with the Berg Balance Scale (BBS) [26]. Vibration sense was measured bilaterally at the medial malleolus and at the first metatarsophalangeal joint with a semi-quantitative tuning fork (Rydel Seiffer, Neurologicals, Poulsbo, Washington [27];). Furthermore, participants executed the Timed Up and Go test (TUG) and the 10-m walking test (10MWT). Additionally, we used this visit to familiarize the participants with the three experimental tasks to reduce the instruction time during the tDCS sessions.

Consecutive tDCS sessions were scheduled with one-week intervals using a cross-over design. In each tDCS session, participants received a different type of tDCS, i.e. anodal stimulation over ipsilesional M1, cathodal stimulation over contralesional M1, or sham stimulation. The order of the different tDCS conditions was balanced across participants. During sham tDCS, the targeted hemisphere was also balanced across participants. Participants were informed that they would receive three different types of tDCS. They were not informed that one tDCS application involved sham stimulation before completion of the last session. Directly after stimulation, participants executed three different movement tasks (as explained below) that all involve the TA as a prime mover. The tasks were designed to be completed within 30 min after tDCS due to the time-limited effects of the stimulation [28, 29]. One participant used an ankle-foot orthosis and two participants used an implanted ankle-dorsiflexion functional electrical stimulation system in daily life; these aids were not used during the experiment.

#### tDCS application

Stimulation was applied with the DC-STIMULATOR PLUS (Neurocom, Illmenau, Germany). Two conductive-rubber electrodes (5x7cm) placed in saline solution-soaked sponges were positioned on the area above the primary motor cortex (C3/C4 of the 10–20 international electro-encephalogram system) and on the contralateral supraorbital region. The stimulation current of 2 mA was applied for 15 min and was ramped up at the start and ramped down at the end of the stimulation over a period of 10 sec. During the sham-session, current was applied two times (at the start and at the end of the 15 min stimulation time) for only 15 s with a ramp up and down period of 10 s. This stimulation protocol mimics the skin sensations as perceived during actual stimulation, but is too short for actual stimulation effects [28]. During the entire stimulation period, participants were instructed to keep an upright stance and not to grasp a table for support.

#### Experimental tasks

Participants performed three movement tasks: ankle dorsiflexion (for the paretic and non-paretic leg separately), gait initiation, and recovering from a backward balance perturbation. These tasks are described in detail below. The primary outcome for all tasks was the reaction time, as measured from electromyographic (EMG) recordings of the TA. Participants performed 12 trials of each task. In the case of the participant being clearly distracted or an obvious false start (as observed by the experimenter), extra trials were added up to a maximum of two. Prior to receiving tDCS, participants performed a few practice trials of each task.

#### Ankle dorsiflexion

Participants sat on a height-adjustable chair in front of two arrays of light-emitting diodes (LEDs; 11 × 8 cm, 3 cm apart) with hip, knees and ankles at a 90° angle. The left array served as a warning sign on which participants needed to prepare the upcoming movement. After a variable interval (1–3.5 s), illumination of the right LED array was the ‘go’ signal on which the participant had to perform an ankle-dorsiflexion movement as fast as possible. The next trial was started by the experimenter as soon as the participant was ready (at least 2 s between trials). Ankle dorsiflexion movements were evaluated for both the paretic and the non-paretic leg in separate blocks. Non-paretic ankle dorsiflexion movements were measured to differentiate between general arousal effects and specific lateralized effects of tDCS.

#### Gait initiation

Participants were standing in front of the LED box at a distance of 2.9 m. Similar to the procedure for the ankle dorsiflexion task, they had to start walking as fast as possible in response to the go-signal by making three steps at a comfortable pace. They were instructed to lead with their preferred stepping leg (same leg as used during the intake session). We chose not to force all participants to use the same leading leg, because we wanted to keep the task as ‘natural’ as possible. This decision was not expected to impact our results, as the TA is activated at similar latencies in both the stance and the stepping leg (albeit with different burst amplitudes) [30]. Furthermore, participants were instructed to keep their weight distribution between both legs as natural as possible and not to move prior to the go-signal, which was checked on-line by the experimenter based on the vertical ground reaction force recordings from two force plates (one under each foot: 60 × 180 cm, AMTI Custom 6 axis composite force platform, USA). The next trial was started by the experimenter as soon as the participant was ready (at least 5 s between trials).

#### Backward balance perturbation

This task involved recovering from a backward loss of balance with a feet-in-place strategy (i.e. without taking a step or grasping handrails for support). Perturbations were delivered on the Radboud Falls Simulator (240 × 174 cm; BAAT, Enschede, The Netherlands) [31] by means of an anterior support-surface translation. The perturbation waveform comprised an acceleration phase of 300 ms at 0.500 m/s^2^, followed by a constant velocity phase of 500 ms, and a deceleration phase of 300 ms. All participants stood with their feet 4.5 cm apart. For safety reasons, participants wore a harness attached to the ceiling to prevent actual falling, and a soft ankle brace (ASO, Medical Specialities, Wadesboro, NC, USA) on the paretic side to prevent possible ankle sprains due to the imposed perturbations. Participants received the balance perturbations with an inter-trial interval varying between 7 and 10 s.

### Data collection

Muscle activity was recorded from bilateral tibialis anterior (TA) at 2000 Hz using surface EMG (ZeroWire, Aurion, Italy) and self-adhesive Ag-AgCl electrodes placed ~ 2 cm apart and on the location as recommended by the SENIAM guidelines [32]. In addition, reflective markers were placed on the heel and the second metatarsal head for determining step onsets during gait initiation; and on the spinous process of the seventh cervical vertebra (C7) for recording body sway during balance perturbations. An additional marker was placed on top of the moveable platform for measuring actual platform movements. Marker trajectories were recorded by an 8-camera 3D motion analysis system (Vicon Motion Systems, United Kingdom) at a sample rate of 100 Hz.

### Data analysis

The EMG signals were band-pass filtered (20–450 Hz, zero-lag, second order Butterworth filter), rectified and low-pass filtered at 30 Hz (zero-lag, second order Butterworth filter).

TA onset latencies were determined using a semi-automatic computer algorithm. TA onset was detected at the instant when muscle activity exceeded baseline activation (defined as the mean muscle activity during 500 ms just prior to the GO-signal + 2 SD). Latencies were visually approved and, if necessary, corrected [33].

Recorded marker trajectories were low-pass filtered at 10 Hz (zero lag, second-order Butterworth filter). Step onset was determined as the instant when the heel or the toe marker exceeded a movement velocity of 0.2 m/s in the anterior direction following the GO signal. Maximum posterior body excursion was determined from the C7 marker trajectory, after subtracting the trajectory of the platform marker. Offline analyses were performed in Matlab R2014b (The MathWorks, Inc., Natick, Massachusetts, United States). To reduce the potential effect of outliers on the higher and lower end of the spectrum, we calculated a truncated mean discarding the two lowest and two highest values of all outcome measures.

### Statistical analyses

To evaluate the effects of tDCS on leg motor output, we conducted repeated measures ANOVAs of all outcome measures. Within-subject factors were tDCS (3 levels: sham vs. anodal vs. cathodal) and leg (2 levels: paretic vs. non-paretic for ankle dorsiflexion and backward balance perturbation), and between-subject factor leg (stepping vs. standing leg for gait initiation). If Mauchly’s test of sphericity was violated, degrees of freedom were corrected by using the Greenhouse-Geisser correction. Tests for normality were run on within-subject differences between tDCS sessions. This showed that within-subject differences were normally distributed for all outcomes, except for the differences in paretic TA and step onset latencies during gait initiation. As non-parametric and parametric statistics yielded similar results and the vast majority of outcomes was normally distributed, we decided to use parametric statistics for all comparisons of interest. To test whether the individual effects of tDCS were associated with the individual degree of leg motor function, Pearson correlation coefficients were determined between the FMA-L scores and the changes (cf. sham stimulation) in each outcome measure following a-tDCS and c-tDCS. The alpha level was set at 0.05. All statistical analyses were performed in IBM SPSS 22 (SPSS, Inc., Chicago, IL, USA).

## Results

Participants’ demographics and clinical characteristics are presented in Table 1. All participants completed the full study protocol. For one participant, paretic TA onset latencies (stance leg) could not reliably be detected during gait initiation, leaving twelve participants for analysis of TA reaction times during gait initiation. All participants reported that they could sense the actual stimulation during all tDCS sessions (including sham). They reported to feel tingling, stabbing or burning sensations with a very subtle to moderate intensity, none of which led to discontinuation of the applied stimulation. The participants did not report any adverse events following the tDCS sessions. After having completed the three tDCS sessions, participants were informed that the protocol involved one sham session. All participants reported to have been unaware of any of the sessions involving sham stimulation.

**Table 1:** Participants’ demographics and clinical characteristics

Age (years)	62 (11.6)
Height (cm)	175.2 (7.8)
Weight (kg)	79.0 (13.7)
BMI	25.7 (3.9)
Gender (M/F)	12/1
Time post stroke (years)	9.2 (6.3)
Paretic side (left/right)	6/7
Type of stroke (ischemic / hemorrhagic)	9/4
Berg Balance Scale [56]	54 (2.0)
FMA-L [34]	24 (6.9)
Motricity Index [33]	
Ankle dorsiflexion	19 (10.7)
Knee extension	26 (4.1)
Hip flexion	27 (4.5)
Vibration Sense [8]	
MTP-1 paretic	5.5 (1.2)
MTP-1 non-paretic	5.7 (1.1)
Medial malleolus paretic	4.8 (2.2)
Medial malleolus non-paretic	5.0 (2.0)
TUG (s)	13.5 (7.7)
10MWT (s)	10.4 (3.5)

Data are presented as ‘mean (standard deviation)’ or number of participants. Maximal scores of clinical assessments are presented between square brackets. *BMI* body mass index, *FMA-L* Fugl-Meyer Assessment – leg score, *MTP-1* first metatarsophalangeal joint, *TUG* Timed Up and Go test, *10MWT* 10-m walking test

### Effects of tDCS on TA onset latencies

**Fig. 1:**
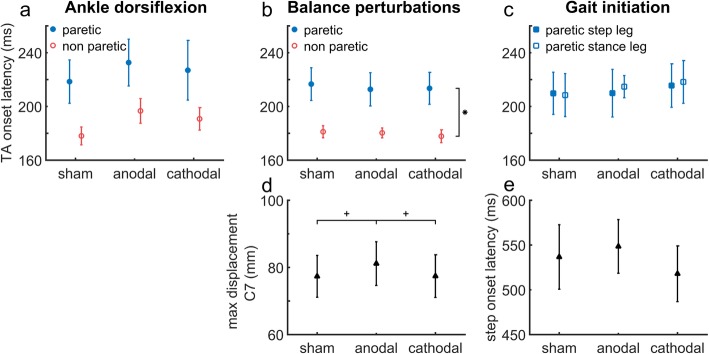
Group average onset latencies (± SE) for the paretic tibialis anterior (TA) for each tDCS session (sham, anodal and cathodal tDCS) for (**a**) ankle dorsiflexion, (**b**) backward balance perturbation and (**c**) gait initiation. Panel d shows C7 displacement (± SE) following balance perturbation for each tDCS session. Panel e displays step onset latencies (± SE) during gait initiation for each session. *Indicates a significant main effect of leg. +Indicates significant differences between tDCS sessions

Figure 1a shows average TA onset latencies for each tDCS condition during ankle dorsiflexion. Onset latencies were not accelerated by either a-tDCS (215 ± 52 ms) or c-tDCS (209 ± 70 ms) compared to sham (198 ± 48 ms; *tDCS*: F_2,24_ = 3.099, *p* = 0.063), which was true for both the (stimulated) paretic leg and the (non-stimulated) non-paretic leg (*tDCS x leg:* F_2,24_ = 0.153, *p* = 0.859). Paretic TA onsets were on average 38 ms slower than non-paretic onsets, which difference was borderline significant (*leg:* F_1,12_ = 4.620, *p* = 0.053). Similarly, following balance perturbations paretic and non-paretic TA onset latencies were not influenced by tDCS (a-tDCS: 197 ± 36 ms; c-tDCS: 196 ± 37 ms; sham: 199 ± 37 ms; *tDCS:* F_2,24_ = 1.629, *p* = 0.217; *tDCS x leg*: F_1.399,16.792_ = 1.071, *p* = 0.358; Fig. 1b). Yet, paretic TA onset latencies were significantly delayed by 35 ms compared to those of the non-paretic leg (*leg:* F_1,12_ = 5.997, *p* = 0.031). During gait initiation tDCS did not influence paretic TA onset latencies either (a-tDCS: 213 ± 43 ms; c-tDCS: 217 ± 53 ms; sham: 209 ± 52 ms; *tDCS:* F_2,20_ = 0.310, *p* = 0.737; Fig. 1c), regardless of whether the paretic or non-paretic leg was used as the leading leg *(tDCS x leg*: F_2,20_ = 0.052, *p* = 0.949; *leg:* F_1,10_ = 0.006, *p* = 0.940).

### Effects of tDCS on body sway and step onset

Following balance perturbations, a small but significant difference between tDCS conditions was found for C7 displacements (*tDCS:* F_2,24_ = 4.216, *p* = 0.027; Fig. 1d). Post-hoc t-tests showed that C7 displacement was slightly larger (4 mm) after a-tDCS compared to sham (t_12_ = − 2.684, *p* = 0.020) and c-tDCS (t_12_ = 2.250, *p* = 0.044). The C7 displacement was not different between c-tDCS and sham stimulation (t_12_ = − 0.048, *p* = 0.963). During gait initiation, neither a-tDCS nor c-tDCS had a significant effect on step onset latencies, regardless of whether the paretic or the non-paretic leg was used as the stepping leg (a-tDCS: 548 ± 108 ms; c-tDCS: 518 ± 112 ms; sham: 537 ± 130 ms; *tDCS:* F_2,22_ = 3.078, *p* = 0.066; *tDCS x leg:* F_2,22_ = 0.902, *p* = 0.420; leg: F_1,11_ = 4.328, *p* = 0.062; Fig. 1e).

### Association between tDCS effects and leg motor function

**Fig. 2:**
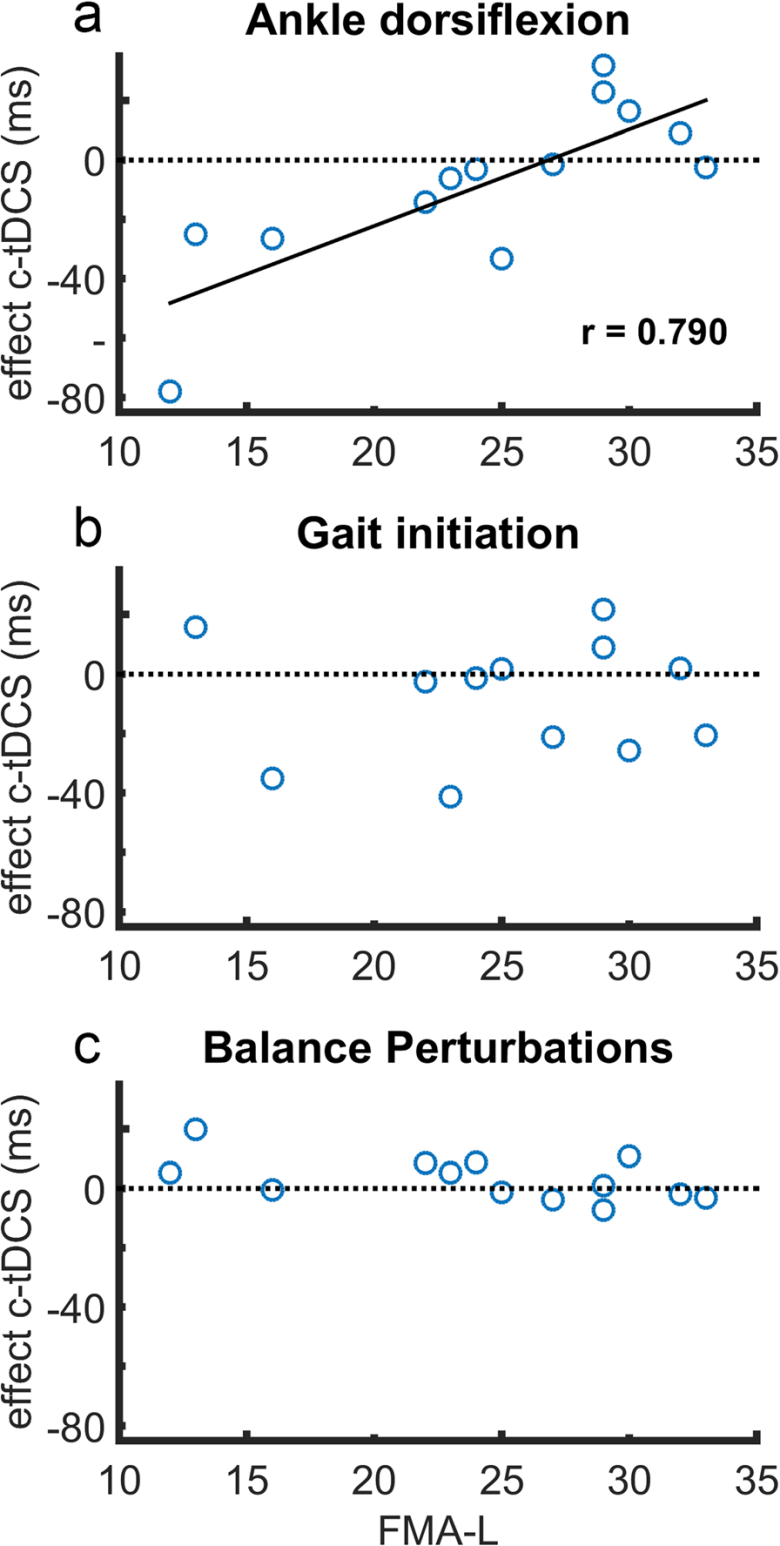
Individual effect of cathodal tDCS relative to a participant’s Fugl-Meyer Assessment –leg score (FMA-L). Effect of c-tDCS is defined as TA onset latency after sham-tDCS minus TA onset latency after c-tDCS. Thus, an effect of > 0 indicates faster onset latencies after c-tDCS

We determined Pearson correlation coefficients between individual effects of tDCS (cf. sham stimulation) and FMA-L scores. For all tasks, we did not observe significant correlations between FMA-L scores and individual effects of a-tDCS on TA onsets, C7 displacements or step onsets (r_p_ = − 0.173 – 0.320). In contrast, the individual effects of c-tDCS on TA reaction times for voluntary paretic ankle dorsiflexion were strongly associated with FMA-L scores (r_p_ = 0.790, *p* = 0.001; Fig. 2a), with more detrimental effects of c-tDCS in patients with poorer leg motor function. Yet, similar associations between FMA-L scores and c-tDCS effects on TA reaction times (Fig. 2b-c), C7 displacements or step onsets were not observed in the gait initiation or backward balance perturbation task (r_p_ = − 0.538 – 0.258).

### Mirror activity during ankle dorsiflexion of the paretic leg

**Fig. 3:**
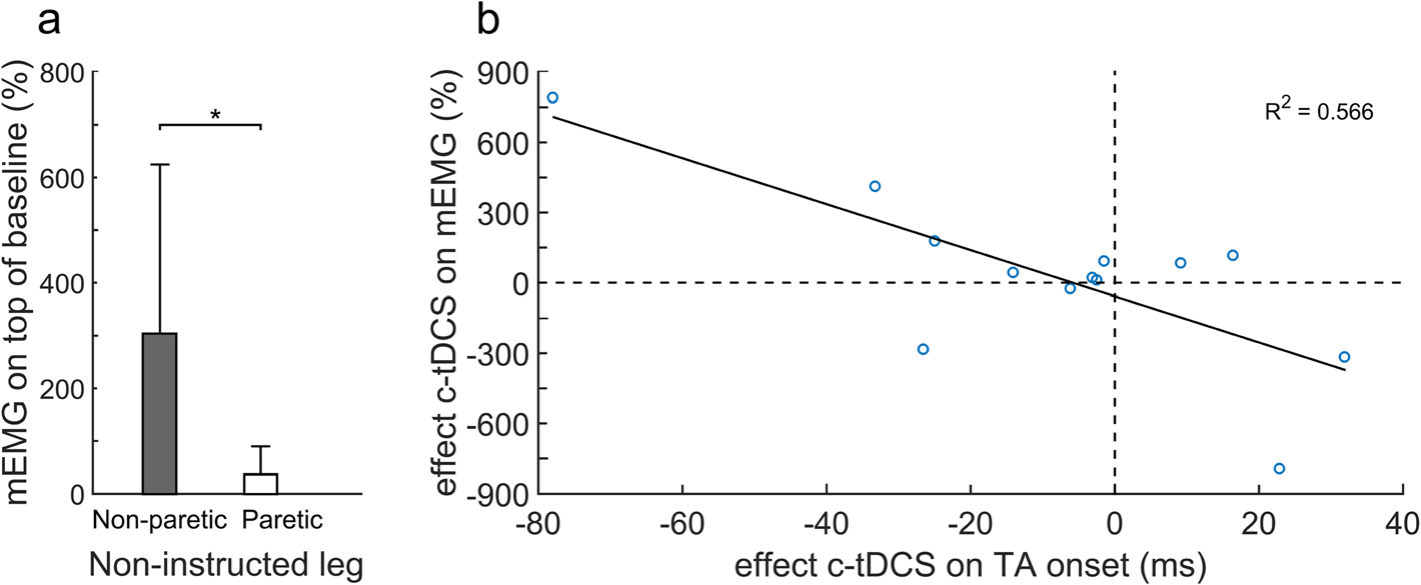
(**a**) Mirror activity (EMG amplitude) in the non-instructed leg as a percentage of baseline activity (mean + SD). A value above 0% indicates a proportional increase in activity compared to baseline. (**b**) Individual effect of c-tDCS on paretic TA onset latency relative to individual effect of c-tDCS on mEMG amplitude in the non-paretic TA. Effect of c-tDCS is defined as outcome of sham-tDCS minus outcome of c-tDCS. Thus, a value > 0 indicates faster onset latencies after c-tDCS. mEMG values of > 0 indicate a decrease of mEMG during c-tDCS

Interestingly, during ankle dorsiflexion of the paretic leg, we observed overt mirror movements of the non-paretic leg in several participants. Therefore, we performed an additional analysis on mirror-EMG (mEMG) activity in the non-instructed leg during ankle dorsiflexion of the other leg. The level of mEMG activity was calculated as the change in amplitude of rectified EMG from baseline (last 500 ms before TA onset) during the first 100 ms post TA onset of the instructed leg. During ankle dorsiflexion of the paretic leg following sham stimulation, we observed > 50% mEMG above baseline in the non-paretic leg of 11/13 participants. Conversely, this was observed in the paretic leg of only 4/13 participants during non-paretic ankle dorsiflexion movements. In addition, the level of mEMG activity during paretic ankle movements was significantly higher compared to the mEMG activity during non-paretic leg movements (268% ± 334%; t_12_ = 2.884, *p* = 0.014; Fig. 3a). Overall, tDCS did not influence mEMG activity. Yet, following c-tDCS, the individual differences in paretic TA onset latencies (cf. sham) showed a strong negative correlation with the individual differences in mEMG latencies (cf. sham) in the non-paretic TA (r_p_ = − 0.753, *p* = 0.003; Fig. 3b).

## Discussion

This study explored whether ipsilesional a-tDCS and/or contralesional c-tDCS may facilitate lower extremity movements in people in the chronic phase after a supratentorial stroke. We studied the effects of offline tDCS on muscle onset latencies in tibialis anterior (TA) as a measure of corticospinal excitability [18, 34] and we used a comprehensive set of tasks that are known to involve early TA recruitment. Overall, we failed to demonstrate significant effects of either a-tDCS or c-tDCS on TA reaction times in any of the tasks. At the individual level, effects of a-tDCS on the paretic leg were not correlated with leg motor function (i.e. Fugl-Meyer Assessment – leg score). In contrast, we observed a strong correlation between leg motor function and individual effects of c-tDCS over contralesional M1 on TA reaction times on the paretic side during voluntary ankle dorsiflexion. We found modest positive effects of c-tDCS in people with good leg motor function and detrimental effects in people who had poor leg motor function after stroke. Remarkably, no such associations were observed for the other tasks.

In contrast to our hypotheses and to previous findings in healthy adults [21, 35], we found no beneficial effects of a-tDCS over the lesioned hemisphere on paretic TA reaction times during a voluntary ankle dorsiflexion task in a group of participants in the chronic phase after stroke. This observation adds to the rather mixed findings reported in the stroke literature, with some studies reporting positive effects of a-tDCS on lower-extremity motor output [13, 14], and other studies demonstrating a lack of such effects [36, 37]. The present study adds to the existing literature by demonstrating that c-tDCS over the contralesional M1 - as a different tDCS application that may indirectly facilitate corticospinal excitability in the stroke-affected hemisphere – did not yield faster TA reaction times in the paretic leg either.

The individual differences that we observed between active tDCS and sham stimulation in our stroke participants were more variable than those observed in our previous study in healthy young individuals (SD = 30 ms vs. 10 ms in Nonnekes et al., [21]), which is a common observation in tDCS studies in the stroke population. The degree of damage to the stimulated area (primary motor cortex) may explain some of the variability in a-tDCS effects, as a previous study found greater a-tDCS effects (as measured from MEPs in paretic first dorsal interosseous) in patients with higher integrity of the (pre)motor cortical network [38]. Similarly, beneficial a-tDCS effects might be expected in patients with better leg motor function (i.e. higher FMA-L scores), but we did not observe such an association. Yet, we did find a strong correlation between the individual effects of c-tDCS on paretic TA reaction times during ankle dorsiflexion and FMA-L scores. The latter finding is in agreement with the results of O’Shea and colleagues [11], who found a similar association when assessing c-tDCS effects on reaction times of paretic hand movements as the primary behavioral outcome. In line with their findings for upper-extremity movements, we found that only participants with good leg motor function (FMA-L scores > 27) experienced modest gains in TA reaction times during ankle dorsiflexion (16 ms on average) following c-tDCS. Such beneficial effects of c-tDCS may indeed be expected based on the interhemispheric inhibition hypothesis [7], which predicts that downregulation of contralesional motor cortical areas leads to gains in motor output from the lesioned side.

In our participants with poor leg motor function, however, the observed detrimental effects of c-tDCS on TA reaction times during ankle dorsiflexion are not in agreement with this hypothesis. Instead, these results may point at this group of patients using their unaffected hemisphere as a ‘back-up’ system for generating motor output to their paretic leg, with c-tDCS downregulating this suggested vicarious activation of the contralesional hemisphere [39]. An additional finding from our study supports this suggestion. In the non-paretic TA, we observed substantial mirror activity during paretic ankle dorsiflexion movements, which is believed to be caused by the contralesional hemisphere trying to contribute to the recruitment of paretic muscle activity but in parallel activates the non-paretic side [39]. As such, downregulation of vicariation from the contralesional hemisphere by c-tDCS would be expected to result in reductions in TA mirror activity, particularly in those patients who demonstrated delayed TA reaction times following c-tDCS during paretic ankle dorsiflexion movements, which is indeed what we observed (see Fig. 3b). Together, our results suggest that c-tDCS over the contralesional M1 may reduce the compensatory recruitment of the contralesional hemisphere in moderately to severely affected stroke patients, thus having a detrimental effect on voluntary motor control of the paretic leg. Possible beneficial effects of c-tDCS appear to be restricted to people with good leg motor function after stroke, who have sufficient ‘structural reserve’ of the lesioned hemisphere [39].

For the backward balance perturbation and gait initiation tasks in our experimental protocol, we neither found any tDCS effects on TA onset latencies at the group level, nor did we observe any association of individual effects with leg motor function. The finding of absent a-tDCS effects on perturbation-induced responses in TA is not in line with our previous study in healthy young participants, in which we used the same stimulation protocol and experimental setup. In that study, we showed a-tDCS to speed up TA onset latencies, albeit only by 7 ms [21]. Several factors, like brain morphology [40] and hormones [41], are suggested to influence the effect of tDCS. Yet, we currently lack good predictors of ‘responders’ vs ‘non-responders’ to tDCS [42]. We are unaware whether these factors may have altered a-tDCS-induced effects differently between our previous and the current study, and may have led to a lack of replication of our previous results. However, similar discrepancies in tDCS effects between people after a stroke and healthy adults have been reported by van Asseldonk and Boonstra, with tDCS resulting in enhanced propulsion during gait in healthy adults, but not in people with stroke [43]. It has been shown that the presence of an ischemic stroke lesion alters the distribution and the maximum value of the electric field induced by tDCS application [44, 45], which may explain the disparate results between healthy persons and people after stroke. Although a-tDCS has been shown to increase MEPs of paretic lower extremity muscles during walking [46], increased corticospinal excitability may not directly translate into gains in balance- and gait-related motor output, because these behaviors are primarily mediated by subcortical pathways [47, 48]. This may also explain why we did not find significant correlations between individual tDCS effects and FMA-L scores.

The lack of beneficial tDCS effects also pertained to the performance-based outcomes (body sway and step initiation time), which findings are in line with other studies that failed to demonstrate such effects of a single tDCS session on balance [49] and gait performance [37, 43] in people after stroke. Although we observed a significant *increase* in body sway following backward balance perturbations in the a-tDCS condition, the difference in C7 excursion compared to the sham condition was a mere 4 mm, which we consider to be of no clinical relevance.

A limitation of our study was the relatively small number of stroke participants (*n* = 13), which resulted in low statistical power (< 0.54 for effects of tDCS on onset latencies) and implies a risk of type II error (i.e. false-negative outcome). It must be mentioned, though, that across tasks and type of tDCS stimulation, TA reaction times were (non-significantly) *delayed* by a mean of 5 ms in the active tDCS conditions compared to sham stimulation. It is, therefore, highly unlikely that a lack of power could explain the absence of significant beneficial tDCS effects in our stroke participants.

## Conclusions

The present findings, albeit obtained from a small group of participants, do not support the use of a single-session of tDCS (at 2 mA with a commonly-used electrode montage over either M1) in the chronic phase after a unilateral supratentorial stroke for improving offline balance and gait performance. However, this notion does not preclude a possible therapeutic potential of *repeated* tDCS sessions as an adjunct to balance or gait training but, thus far, studies on such repeated tDCS applications have shown inconsistent results [3, 20]. Future studies may focus on the question whether different tDCS montages (e.g. stimulation intensity and electrode location) based on personalized models (derived from structural MRI scans) that take into account individual lesion characteristics and maximize current density in the brain areas of interest [45] yield more consistent effects. Such studies are needed to address the question whether tDCS does or does not have any added value to the current rehabilitation treatment for improving balance and gait after stroke.

## Data Availability

The datasets used and/or analyzed during the current study are available from the corresponding author on request.

## Abbreviations

10MWT: 10-m walking test
ARAT: Action Research Arm Test
a-tDCS: anodal transcranial direct current stimulation
BBS: Berg Balance Scale
C7: seventh cervical vertebra
c-tDCS: cathodal transcranial direct current stimulation
EMG: Electromyography
FMA-L: Fugl-Meyer Assessment – leg score
LED: Light-emitting diode
M1: Primary motor cortex
m-EMG: Mirror-electromyography
MEP: Motor evoked potential
MRI: Magnetic Resonance Imaging
TA: Tibialis anterior
tDCS: transcranial direct current stimulation
TUG: Timed Up and Go test
